# Supplementary material for: Mechanisms Underlying the Rhizosphere-To-Rhizoplane Enrichment of Cellvibrio Unveiled by Genome-Centric Metagenomics and Metatranscriptomics
Source: Microorganisms. 2020 Apr 17;8(4):583. doi: 10.3390/microorganisms8040583 (PMC7232360; doi:10.3390/microorganisms8040583)
Supplement: Supplementary file 1 [file microorganisms-08-00583-s001.zip › supplementary files Table S1, S2 and Fig. S1.pdf]

Table S1. Relationship between the metagenome-assembled genome *Cellvibrio* sp. Bin79 and related genomes

Query genome	Reference genome	DDH	Model C.I.	Bootstrap C.I.	Distance	Prob. DDH >= 70%	ANI
**bin.79**	***Cellvibrio japonicus* Ueda107**	**19.8**	**[17.6 - 22.3%]**	**19.8 - 19.9%**	**0.2215**	**0**	**77.72**
**bin.79**	***Cellvibrio* sp. BR**	**19.6**	**[17.4 - 22%]**	**19.6 - 19.6%**	**0.2238**	**0**	**78.50**
**bin.79**	***Cellvibrio mixtus* sp. mixtus J3-8**	**24.6**	**[22.2 - 27%]**	**24.5 - 24.6%**	**0.1776**	**0.01**	**82.84**
**bin.79**	***Cellvibrio* sp. OA-2007**	**19.4**	**[17.2 - 21.8%]**	**19.4 - 19.4%**	**0.2268**	**0**	**78.82**
**bin.79**	***Cellvibrio* pealriver PR1**	**20.6**	**[18.4 – 23%]**	**20.6 – 20.6%**	**0.2135**	**0**	**79.25**

Notes: The DDH and ANI values were determined using the GGDC 2.1 server and fastANI.

Table S2. Identified glycoside hydrolases (GHs) in *Cellvibrio* sp. Bin79 and related genomes

GHs	***Cellvibrio* sp. Bin79**	***Cellvibrio japonicus* ueda107**	***Cellvibrio* sp. BR**	***Cellvibrio* mixtus J3-8**	***Cellvibrio* sp. OA-2007**	***Cellvibrio* Pealriver PR1**
**GH1**	3	1	1	2	2	2
**GH10**	6	4	4	4	5	3
**GH100**	1	0	1	1	0	0
**GH103**	3	3	3	3	3	4
**GH105**	1	1	6	2	3	3
**GH106**	3	3	3	3	3	3
**GH108**	0	0	0	2	0	0
**GH109**	5	5	6	5	5	5
**GH11**	5	2	2	4	2	2
**GH110**	1	0	2	1	1	0
**GH115**	1	1	2	1	1	1
**GH117**	11	6	12	6	7	11
**GH119**	3	0	1	1	2	4
**GH121**	4	2	1	0	1	0
**GH127**	2	1	1	1	1	1
**GH128**	2	2	1	2	1	1
**GH13**	11	17	10	12	10	10
**GH130**	4	3	7	4	6	5
**GH133**	2	0	1	1	1	0
**GH134**	0	0	0	0	1	0
**GH15**	3	3	4	2	3	3
**GH16**	10	9	8	10	9	12
**GH18**	9	4	2	7	4	8
**GH19**	2	1	1	1	1	4
**GH2**	3	4	4	5	5	5
**GH20**	3	2	2	4	2	3
**GH23**	9	6	8	8	7	7
**GH24**	0	0	1	0	0	1
**GH25**	0	0	1	0	0	0
**GH26**	5	3	3	2	5	4

**GH27**	1	1	1	1	1	1
**GH28**	1	1	4	1	1	2
**GH29**	0	0	1	0	0	0
**GH3**	7	4	3	9	7	4
**GH30**	2	2	1	2	1	1
**GH31**	1	3	0	1	1	1
**GH32**	4	5	15	3	4	7
**GH33**	1	2	1	0	1	0
**GH35**	2	3	3	2	2	1
**GH36**	1	1	1	1	1	1
**GH37**	1	2	1	1	0	1
**GH38**	1	0	0	0	0	0
**GH39**	1	3	2	0	2	2
**GH4**	0	1	0	0	0	0
**GH42**	3	3	8	3	6	7
**GH43**	17	16	26	13	15	19
**GH44**	1	0	0	0	0	0
**GH45**	1	1	0	1	0	0
**GH46**	0	1	0	0	0	0
**GH49**	2	0	3	0	0	0
**GH5**	21	20	16	17	22	12
**GH50**	0	0	3	0	2	3
**GH51**	2	2	1	2	1	1
**GH53**	2	3	2	2	2	1
**GH55**	1	0	1	0	0	1
**GH57**	1	1	0	1	1	1
**GH59**	0	0	1	0	0	0
**GH6**	4	1	1	0	1	0
**GH62**	2	1	2	1	1	0
**GH63**	1	2	1	1	0	1
**GH67**	1	1	1	1	1	1
**GH68**	0	0	1	0	1	1
**GH70**	0	2	1	0	0	1
**GH72**	1	1	1	0	0	0
**GH73**	3	2	2	2	3	2
**GH74**	1	3	3	1	1	1
**GH76**	4	1	2	3	2	2
**GH77**	2	1	0	2	1	1
**GH78**	5	4	4	5	2	3
**GH81**	2	1	2	1	1	4
**GH82**	1	1	2	1	1	1
**GH84**	1	1	1	1	1	1
**GH85**	2	0	0	0	0	2
**GH86**	1	1	2	0	2	2
**GH87**	12	4	3	8	4	10
**GH88**	0	0	1	0	0	1
**GH9**	5	3	4	8	4	4
**GH91**	1	0	0	1	1	0
**GH93**	0	2	1	0	0	0
**GH94**	2	1	3	2	1	1
**GH95**	2	2	3	2	2	2
**GH97**	4	3	4	2	2	3
**GH98**	0	1	0	0	0	0
**GH99**	1	0	0	0	0	0

**Total genes**	244	197	237	196	193	212

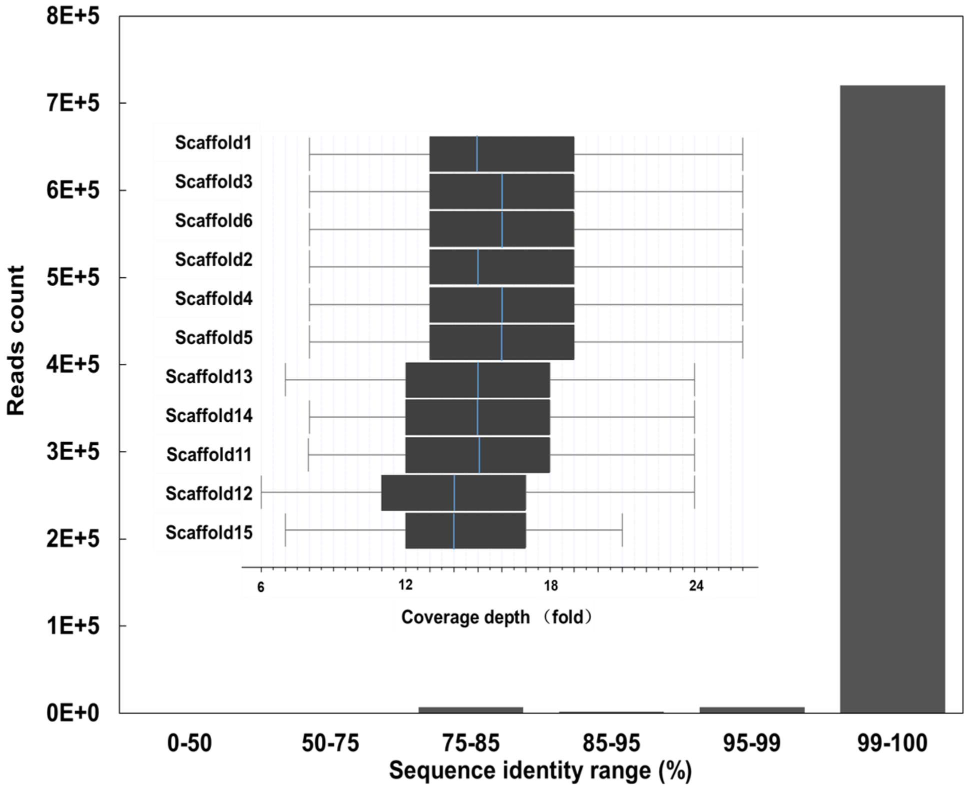

Fig. S1. Quality of the *Cellvibrio* sp. Bin79 genome assembly revealed by sequence identity between metagenomic reads and the genome sequences and genome coverage. The metagenomic reads from sample 2Pm were used. Sequence coverage is shown as a boxplot and the blue line indicates the median value.
